# Whole Exome Sequencing Uncovered the Genetic Architecture of Growth Hormone Deficiency Patients

**DOI:** 10.3389/fendo.2021.711991

**Published:** 2021-09-13

**Authors:** Chenxi Yu, Bobo Xie, Zhengye Zhao, Sen Zhao, Lian Liu, Xi Cheng, Xiaoxin Li, Bingyan Cao, Jiashen Shao, Jiajia Chen, Hengqiang Zhao, Zihui Yan, Chang Su, Yuchen Niu, Yanning Song, Liya Wei, Yi Wang, Xiaoya Ren, Lijun Fan, Beibei Zhang, Chuan Li, Baoheng Gui, Yuanqiang Zhang, Lianlei Wang, Shaoke Chen, Jianguo Zhang, Zhihong Wu, Chunxiu Gong, Xin Fan, Nan Wu

**Affiliations:** ^1^Department of Orthopedic Surgery, Peking Union Medical College Hospital, Peking Union Medical College and Chinese Academy of Medical Sciences, Beijing, China; ^2^Beijing Key Laboratory for Genetic Research of Skeletal Deformity, Beijing, China; ^3^Department of Joint Surgery, Shandong Provincial Hospital Affiliated to Shandong First Medical University, Jinan, China; ^4^Department of Pediatrics, The Second Affiliated Hospital of Guangxi Medical University, Guangxi, China; ^5^Center for Medical Genetics and Genomics, The Second Affiliated Hospital of Guangxi Medical University, Guangxi, China; ^6^Department of Pediatric Endocrine and Metabolism, Maternal and Child Health Hospital of Guangxi, Nanning, China; ^7^Medical Research Center, Peking Union Medical College Hospital, Peking Union Medical College and Chinese Academy of Medical Sciences, Beijing, China; ^8^Department of Endocrinology, Genetics and Metabolism, Beijing Children’s Hospital, National Center for Children’s Health, Capital Medical University, Beijing, China; ^9^Department of Orthopaedic Surgery, Qilu Hospital, Cheeloo College of Medicine, Shandong University, Jinan, China; ^10^Key Laboratory of Big Data for Spinal Deformities, Chinese Academy of Medical Sciences, Beijing, China; ^11^State Key Laboratory of Complex Severe and Rare Diseases, Peking Union Medical College Hospital, Chinese Academy of Medical Science and Peking Union Medical College, Beijing, China

**Keywords:** growth hormone deficiency, whole exome sequencing, genetic architecture, molecular diagnosis, mutational burden analysis

## Abstract

**Purpose:**

Congenital growth hormone deficiency (GHD) is a rare and etiologically heterogeneous disease. We aim to screen disease-causing mutations of GHD in a relatively sizable cohort and discover underlying mechanisms *via* a candidate gene-based mutational burden analysis.

**Methods:**

We retrospectively analyzed 109 short stature patients associated with hormone deficiency. All patients were classified into two groups: Group I (n=45) with definitive GHD and Group II (n=64) with possible GHD. We analyzed correlation consistency between clinical criteria and molecular findings by whole exome sequencing (WES) in two groups. The patients without a molecular diagnosis (n=90) were compared with 942 in-house controls for the mutational burden of rare mutations in 259 genes biologically related with the GH axis.

**Results:**

In 19 patients with molecular diagnosis, we found 5 possible GHD patients received known molecular diagnosis associated with GHD (*NF1* [c.2329T>A, c.7131C>G], *GHRHR* [c.731G>A], *STAT5B* [c.1102delC], *HRAS* [c.187_207dup]). By mutational burden analysis of predicted deleterious variants in 90 patients without molecular diagnosis, we found that *POLR3A* (*p* = 0.005), *SUFU* (*p* = 0.006), *LHX3* (*p* = 0.021) and *CREB3L4* (*p* = 0.040) represented top genes enriched in GHD patients.

**Conclusion:**

Our study revealed the discrepancies between the laboratory testing and molecular diagnosis of GHD. These differences should be considered when for an accurate diagnosis of GHD. We also identified four candidate genes that might be associated with GHD.

## Introduction

Congenital growth hormone deficiency (GHD) is a rare disease characterized by decreased growth hormone (GH) secretion of the anterior pituitary, which leads to growth impairment and metabolic dysfunction in children (1, 2). The causes of GHD include pituitary dysplasia and pathogenic mutations in GH-insulin like growth factor 1 (IGF1) axis-related genes, such as *GH1*, *GHRHR* (3, 4). However, recent cohort studies of the genetic causes of GHD patients in South and East Asian populations reported that the proportion of patients with molecular diagnosis ranges from 4% to 43% (5, 6). The undiagnosed rate indicates that there are still a lot of unknowns about the genetic predispositions of GHD. It is challenging to diagnose GHD only based on clinical symptoms. Clinical presentation of symptoms including neonatal hypoglycemia, midfacial defects such as cleft lip or palate, history of external head injuries, and vacuolated Sella turcica found in pituitary magnetic resonance imaging (MRI) or pituitary dysplasia can assist in GHD diagnosis (7–9). According to the guidelines set by the GH Research Society (GRS), the clinical diagnosis of GHD needs to be based on growth and development data, and data from various laboratory tests (i.e. decreased peak of GH provocation test with two different agents, serological detection of IGF1 and IGF1BP3, combined gonadal hormone when necessarily, and genetic test), and imaging data (craniocerebral MRI) (10). However, there is still a high false-positive rate in the existing serological diagnosis methods (11–14).

With the application of whole exome sequencing (WES) in the exploring the etiology of GHD, an increasing number of GHD-associated genes have been discovered (9). It has been recently reported that a variety of congenital diseases associated with GHD are caused by certain genetic variants. For example, choreoathetosis, hypothyroidism, and neonatal respiratory distress caused by *NKX2-1* (MIM: 610978), neurofibromatosis, type 1 was caused by *NF1* (MIM: 162200), growth plate disorders caused by *FLNB* (15–17). To further explore the contribution of genetic variations to GHD and comprehensively decipher the molecular basis of GHD at an exome level, we herein investigated the difference between molecular findings and clinical diagnosis criteria by whole exome sequencing (WES) data in a cohort of 109 possible GHD patients. To elucidate the genetic architecture of GHD patients, we further analyzed the mutational spectrum in patients without a molecular diagnosis by a mutational burden analysis.

## Materials and Methods

### Cohort Collection

From July 2014 to August 2018, we screened 561 patients with short stature from three centers in China [Maternal and Child Health Hospital of Guangxi Zhuang Autonomous Region, the Second Affiliated Hospital of Guangxi Medical University and Beijing Children’s Hospital, all three centers as parts of the DISCO study (http://www.discostudy.org/)]. Patients with pituitary tumors, chronic diseases, iatrogenic shortness, and previous GH treatment were excluded from this cohort. Demographic information, physical examination results and clinical symptoms were taken into account. Details of cohort were reported in the previous study (18, 19).

Radiographic evaluation included conventional radiographs of the anterior-posterior view of the left hand and pituitary MRI. Radiographs of the left hand and wrist for bone age were assessed independently by two pediatric clinicians. GH stimulation tests with L-dopa and insulin were performed. L-dopa was administered orally, and insulin was subcutaneously injected in the overnight fasting state. Blood samples were collected at 0, 30, 60, 90, and 120 min, respectively. GH concentration of each time was measured using a chemiluminescence method with a sensitivity of 0.01 ng/ml. Serum IGF1 was measured by the chemiluminescence immunometric method. Two GH stimulation tests were provided for all enrolled patients. This study was obtained approval from the ethics committee at the Maternal and Child Health Hospital of Guangxi Zhuang Autonomous Region (G-1-1), the Second Affiliated Hospital of Guangxi Medical University (2020-KY[0112]) and Beijing Children’s Hospital (Y-028-A-01).

**Figure 1:**
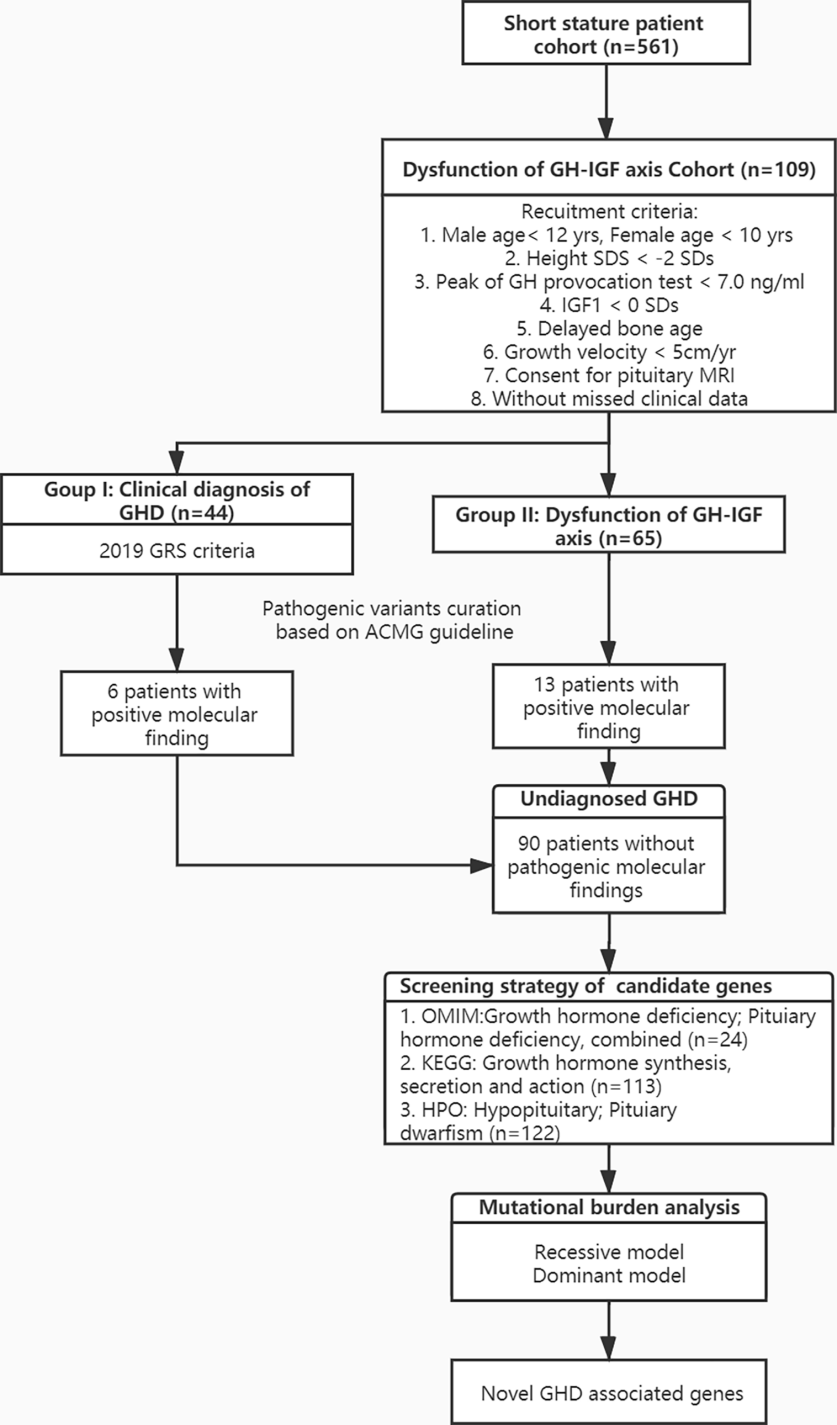
Flowchart of patient enrollment and genetic testing. GHD, growth hormone deficiency; GH, growth hormone; IGF, insulin like growth factor 1; SD, standard deviation; OMIM, Online Mendelian Inheritance in Man; KEGG, Kyoto Encyclopedia of Genes and Genomes; HPO, Human Phenotype Ontology; GRS, GH Research Society.

Based on GHD diagnostic criteria of other countries and reference data of serology in the East Asian populations (20, 21), we recruited 109 unrelated patients with potential GHD using following eligibility criteria: 1) age of male patient < 12 yrs., female patient age < 10 yrs., 2) height standard deviation < -2 standard deviation (SDs), 3) the peak GH concentration < 7ng/ml in one stimulation test, 4) the standard deviation of IGF1 serum level < 0 SDs, 5) the anterior-posterior plain radiograph of left wrist and hand showed delayed bone age, 6) growth velocity < 5cm/yr, 7) availability of MRI examination of the pituitary gland. According to the latest diagnostic criteria of GRS in 2019 (10), all enrolled patients were grouped according to the following items: (1) the peak GH concentration < 7ng/ml in both provocation tests, (2) the standard deviation of IGF1 serum level < -2 SDs, (3) the delayed bone age was assessed independently by two different doctors. Recruited patients were further divided into two groups: Group I (n=45) included patients considered to have definitive GHD by currently stringent diagnosis; Group II (n=64) included patients with possible GHD (**Figure 1**).

### Whole-Exome Sequencing

DNA samples from three centers were processed and prepared under the same protocol from patient blood. In total, 109 patients underwent proband-only WES, while 9 underwent trio-based WES (total 127 subjects) (**Supplementary Methods**). The in-house control database consisted of WES data from 942 unrelated Chinese individuals with no apparent skeletal anomalies and endocrinal distortion from the DISCO project (http://www.discostudy.org/).

### Variant Calling and Interpretation

The sequencing data were analyzed and annotated using an in-house developed analytical pipeline, the Peking Union Medical College hospital Pipeline (PUMP) (22–24) (**Supplementary Methods**). We performed an agnostic analysis of WES data for causal variants potentially contributing to individual patients’ clinically observed phenotypes. The interpretation of all quality controlled and annotated variants was based on American College of Medical Genetics and Genomics (ACMG) guidelines (25) (**Supplementary Methods**).

### Variant Validation and Sanger Sequencing

All pathogenic and likely pathogenic variants were manually reviewed using Integrative Genomics Viewer (IGV) (26). Sanger sequencing was performed independently on available subjects and parental samples to validate variant interpretation by an orthogonal sequencing method and to investigate segregation according to Mendelian expectations for the identified variant allele(s).

### Mutational Burden Analysis

To extend the spectrum of known mutations in genes contributing to GH synthesis and secretion, we performed mutational burden analysis to search for rare variants of uncertain significance (VUS) in 259 candidate genes in the WES data. We first selected 259 candidate genes related to the synthesis and secretion of GH according to the Human Phenotype Ontology (HPO, https://hpo.jax.org/), the OMIM (http://omim.org/) and the Kyoto Encyclopedia of Genes and Genomes (KEGG,https://www.genome.jp/kegg/) databases (**Table S1**).

The variants filtering criteria was based on dominant model and recessive model according to the allele mutation site. The filtering criteria of the dominant model for predicted pathogenic mutations include: 1) public database frequency was required to be < 10^-4^; 2) The missense mutations with Combined Annotation Dependent Depletion (CADD) score > 20 were considered as high credibility; 3) truncating mutations; 4) intra-frame mutations require a range of influence of more than one amino acid; 5) splicing mutations include canonical splicing mutations or mutations with a spliceAI predictions score more than 0.5 (27). For the recessive model, we required that the public database frequency be less than 10^-3^, and the rest of the criteria are the same as the dominant model.

### Statistical Analysis

Mean comparison of relevant features was conducted using Student’s t-test. One-tailed *p*-values of < 0.05 were considered statistically significant. Fisher’s exact test and chi-square test were used for genetic burden analysis.

## Results

### Cohort Information and Clinical Characteristics

A total of 109 unrelated patients were enrolled from previous cohort, including 9 cases with un-affected parents (trios) and 100 singletons (total 127 subjects). Of the 109 patients, 87 were male and 22 were female, with a mean age of 7.6 ± 3.0 years. According to the diagnostic criteria, all patients were divided into two groups. Group I consisted of 44 patients (8 female and 36 male, average age 8.6 ± 2.7) and group II of 65 patients (14 female and 51 male, average age 7.0 ± 3.0) (**Table 1**). There were no significant differences in sex-distribution and delayed bone age between Group I and II except for age, Z-score of height, peak GH concentration, IGF1 and growth velocity (**Table 1**).

**Table 1:** Demographic and clinical characteristic of GHD cohort.

Parameter	Group I (n = 44)	Group II (n = 65)	*p* value	Total (n = 109)
**Age in years, mean (SDs)**	8.6 (2.7)	7.0 (3.0)	0.004	7.6 (3.0)
**Sex, N (%)**				
Male	36 (82%)	51 (79%)	0.67	87 (79.8%)
Female	8 (18%)	14 (22%)		22 (20.2%)
**Height, Z score, mean (SDs)**	-4.2 (1.7)	-3.2 (1.0)	0.002	-3.6 (1.4)
Peak GH concentration in provocation test*, ng/ml, mean (SDs)	2.3 (2.2)	4.7 (1.7)	<0.001	3.7 (2.3)
**IGF1, Z score, mean (SDs)**	-2.3 (0.4)	-1.2 (0.6)	<0.001	-1.6 (0.7)
**Delayed bone age, years (SDs)**	-2.5 (1.4)	-2.1 (1.2)	0.12	-2.3 (1.3)
**Growth velocity, cm/yrs (SDs)**	3.8 (0.9)	4.2 (0.7)	0.04	4.0 (0.8)

*GH provocation tests were performed by both L-dopa and insulin methods. T-test and Chi square test were used for calculation of p value. P < 0.05 was consider as statistical significance. SD, standard deviation.

### Spectrum of Causal Gene and Variants

After WES and variant interpretation based on ACMG guidelines (25), causal variants in 14 genes were identified in 19/109 (17.4%) of the patients. Of 14 genes, seven genes were associated with GH secretion and synthesis, and 10 variants in these genes were identified in patients from both groups (**Table 2**). Interestingly, DISCO-S693 of group I and DISCO-S255 of group II harbored distinct variants in *GHRHR* and had different IGF1 serum levels (-1.61 SDs for DISCO-S255, -2 SDs for DISCO-S693), with only DISCO-S693 meeting stringent diagnostic criteria (**Table 2**). Therefore, we speculated that the adoption of strict clinical diagnostic criteria, particularly IGF1 being less than -2 SDs, could lead to a certain false-negative rate in the diagnosis of GHD.

**Table 2:** Clinical information of patients with positive molecular diagnosis.

ID	Group	Gender	CA, yrs	Height SDS	PH SDs	MH SDs	Delayed BA, yrs	IGF1 SDS	Peak of GPT, ng/ml	Pituitary MRI	Causal gene	Molecular diagnosis	Inheritance	Zygosity	Reference sequence	Genomic position#	cDNA change	Protein change	Mutation type	Origin	CADD score	Additional phenotype
DISCO-S049	II	M	11.00	-4	-4.47	0.07	-0.50	-1.74	5.94	No obvious abnormality	*ACAN*	Short Stature and Advanced Bone Age, with or without Early-onset Osteoarthritis and/or Osteochondritis Dissecans (MIM: #165800)	AD	Het	NM_013227.3	chr15:89388801	c.1117_1120delCAGA	p.Thr374fs	Frameshift	NA	NA	NA
DISCO-S028	II	F	7.67	-7	-1.24	-1.96	-5.17	-1.00	5.38	No obvious abnormality	*COL11A1*	Marshall Syndrome (MIM: #154780)	AD	Het	NM_001854.3	chr1:103381186	c.3816+1G>A	–	Splicing	*de novo*	NA	Depressed nasal bridge HP:0005280; Long philtrum HP:0000343
DISCO-S051	I	F	5.25	-5	-1.56	-3.26	-1.75	-2.00	1.41	No obvious abnormality	*GH1*	Growth Hormone Deficiency (MIM: #173100)	AD	Het	NM_000515.4	chr17:61995746	c.131A>C	p.His44Pro	Missense	Maternal	13.79	NA
DISCO-S594	I	M	11.92	-3.7	-3.02	-0.67	-2.92	-2.56	2.06	Small pituitary gland	*GH1*	Growth hormone deficiency, isolated, type II (MIM: #173100)	AD	Het	NM_000515.4	chr17:61994697	c.626G>A	p.Arg209His	Missense	NA	NA	NA
DISCO-S255	II	M	3.75	-5.5	-2.05	-1.78	-1.75	-1.61	0.94	The adenohypophysis was significantly reduced, and the subarachnoid herniated into the sella	*GHRHR*	Growth Hormone Deficiency, Isolated, Type IV (MIM: #618157)	AR	Hom	NM_000823.3	chr7:31013733	c.731G>A	p.Trp244Ter	Nonsense	NA	36	Micrognathia HP:0000347; Depressed nasal ridge HP:0000457; Anteverted nares HP:0000463; Protruding ear HP:0000411
DISCO-S693	I	M	5.17	-4.69	-0.92	-1.04	-1.67	-2.00	0.52	Small pituitary gland	*GHRHR*	Growth Hormone Deficiency, Isolated, Type IV (MIM: #618157)	AR	Com Het	NM_000823.3	chr7:31011594, chr7:31016171	c.481C>T, c.1102C>T	p.Arg161Trp, p.Gln368Ter	Missense, Nonsense	Com Het	16.53, 39	Abnormality of the pituitary gland HP:0012503
DISCO-S071	II	M	7.10	-2.9	-0.76	0.44	-1.60	-1.84	3.94	Larger and deeper sella, thin pituitary gland, Chiari deformity, and shorter and smaller corpus callosum	*HRAS*	Costello syndrome (MIM: #218040)	AD	Het	NM_005343.2	chr11:533848	c.187_207dup	p.Glu63_Asp69dup	Inframe insertion	*de novo*	NA	Intellectual disability HP:0001249; Motor delay HP:0001270; Strabismus HP:0000486; Depressed nasal bridge HP:0005280; Low-set ears HP:0000369; Low posterior hairline HP:0002162; Nevus HP:0003764; Acanthosis nigricans HP:0000956; Clubbing of fingers HP:0100759; Arnold-Chiari malformation HP:0002308; Short corpus callosum HP:0200012
DISCO-S032	II	M	6.00	-2.2	0.21	-0.48	-1.00	-1.53	1.40	No obvious abnormality	*IDS*	Mucopolysaccharidosis, Type II (MIM: #309900)	XLR	Hem	NM_000202.6	chrX:148585007	c.253G>A	p.Ala85Thr	Missense	NA	NA	NA
DISCO-S189	I	M	3.75	-3.51	-0.11	0.81	-1.75	-2.00	0.78	Pituitary stalk block syndrome	*IDS*	Mucopolysaccharidosis Type II (MIM: #309900)	XLR	Hem	NM_000202.6	chrX:148585007	c.253G>A	p.Ala85Thr	Missense	NA	NA	Abnormality of the hypothalamus-pituitary axis HP:0000864; Strabismus HP:0000486
DISCO-S607	I	M	9.20	-3.73	-1.65	-1.96	-1.70	-2.48	4.22	Small pituitary gland	*MAP2K1*	Cardiofaciocutaneous syndrome 3 (MIM: #615279)	AD	Het	NM_002755.3	chr15:66729219	c.427A>G	p.Met143Val	Missense	Maternal	23.8	NA
DISCO-S608	II	M	5.00	-2.89	0.05	-0.30	-3.00	-0.53	3.91	No obvious abnormality	*MMP13*	Spondyloepimetaphyseal dysplasia, Missouri type (MIM: #602111)	AD	Het	NM_002427.3	chr11:102826120	c.223T>C	p.Phe75Leu	Missense	NA	20.7	NA
DISCO-S289	II	M	4.50	-4.5	-1.24	-1.04	-3.00	-1.26	3.01	Small pituitary gland, Subcerebrospinal fluid hernia	*NF1*	Neurofibromatosis, Type I (MIM: #162200)	AD	Het	NM_000267.3	chr:29554544	c.2329T>A	p.Trp777Arg	Missense	*de novo*	22.2	Ptosis HP:0000508; Low-set ears HP:0000369; Cafe-au-lait spot HP:0000957; Spina bifida occulta at L5 HP:0004601
DISCO-S179	II	M	8.00	-2.66	-0.11	0.81	-2.50	-1.34	6.87	No obvious abnormality	*NF1*	Neurofibromatosis, Type I (MIM: #162200)	AD	Het	NM_000267.3	chr17:29676142	c.7131C>G	p.Tyr2377Ter	Nonsense	NA	14.38	NA
DISCO-S344	II	M	3.08	-3.1	-0.44	-0.48	-1.08	-0.54	4.54	Small pituitary gland	*PTPN11*	Noonan Syndrome 1 (MIM: #163950)	AD	Het	NM_002834.3	chr12:112888220	c.236A>G	p.Gln79Arg	Missense	*de novo*	28.5	Atrial septal defect HP:0001631; Microcephaly HP:0000252; Micrognathia HP:0000347; Depressed nasal bridge HP:0005280; Hyperplasia of midface HP:0012371
DISCO-S167	II	M	6.17	-2.68	-2.37	-0.11	-2.17	-1.74	2.14	No obvious abnormality	*PTPN11*	Noonan Syndrome 1 (MIM: #163950)	AD	Het	NM_002834.3	chr12:112910835	c.844A>G	p.Ile282Val	Missense	*de novo*	17.88	NA
DISCO-S186	II	M	8.67	-2.82	-2.68	-0.67	-3.67	-0.75	5.78	No obvious abnormality	*ROR2*	Brachydactyly, type B1 (MIM: #113000)	AD	Het	NM_004560.3	chr9:94486150	c.2625dupC	p.Thr876fsTer20	Frameshift	Paternal	NA	NA
DISCO-S232	I	F	10.67	-8	-2.05	-2.33	-4.17	-3.25	5.20	No obvious abnormality	*SLC7A7*	Lysinuric Protein Intolerance; LPI (MIM: #222700)	AR	Com Het	NM_001126106.2	chr14:23243183, chr14:23243580	c.1387delG, c.1228C>T	p.Val463CysfsTer56, p.Arg410Ter	Frameshift, Nonsense	Com Het	NA, 38	Spina bifida occulta at S1 HP:0004614; Hypertelorism HP:0000316; Malaligned philtral ridges HP:0011827; Intellectual disability HP:0001249; Narrow forehead HP:0000341; Depressed nasal bridge HP:0005280
DISCO-S041	II	M	10.00	-2.6	-1.73	-1.41	-2.00	-0.23	4.53	No obvious abnormality	*STAT5B*	Growth hormone insensitivity with immunodeficiency (MIM: #245590)	AD	Het	NM_012448.3	chr17:40370236	c.1102delC	p.Gln368fsTer2	Frameshift	NA	NA	NA
DISCO-S334	II	M	4.92	-3	-0.44	-0.67	-2.92	-1.71	6.84	No obvious abnormality	*TRPS1*	Trichorhinophalangeal Syndrome, Type I (MIM: #190350)	AD	Het	NM_014112.4	chr8:116632231	c. 94C>T	p.Gln32Ter	Nonsense	NA	29.1	NA

#Genomic position was based on GRCh37. CA, Chronological Age; PH, Paternal Height; MH, Maternal height; BA, Bone Age; GPT, GH provocation test; F, female; M, male; NA, not applicable. Het, heterozygous; Hom, homozygous; Hem, hemizygous; Com Het, compound heterozygous; AD, autosomal dominant; AR, autosomal recessive; XLR, X-linked recessive.

Interestingly, seven genes that were not previously associated with GHD were identified in 9 patients from both groups. Same variants (c.253G>A[p.Ala85Thr]) in *IDS* were identified in 2 patients, patient DISCO-S189 from Group I and patient DISCO-S032 from Group II (**Table 2**). Seven patients carried pathogenic or likely pathogenic variants in 7 genes which had no clear evidence supporting their biological function in GH synthesis or secretion (**Table 2**, **Figure S1**). Our results showed that patients with GHD might have defects in gene expression relating to diverse biological functions.

### Genetic Burden Analysis Revealed Novel Genes Biological Related to GH Axis

Due to the discrepancy between the clinical diagnosis and molecular diagnosis in GHD patients, the adoption of less stringent inclusion criteria might help explore the molecular mechanism of GHD more comprehensively. To this end, we compared the mutational burden of rare coding variants of each candidate gene between the patients without a molecular diagnosis and the control samples. Among all the genes related to GH synthesis and secretion, we found that rare VUS in 40 genes enriched in our GHD cohort (**Table S2**). Among 40 genes enriched in our cohort, four genes were found with trend toward significance: *POLR3A* (*p* = 0.005), *SUFU* (*p* = 0.006), *LHX3* (*p* = 0.021), *CREB3L4* (*p* = 0.040) (**Tables 3** and **S2**).

**Table 3:** Genetic burden analysis for rare VUS in GHD cases and controls.

Gene symbol	Variant alleles (n = 90)	Variant alleles (n = 942)	*P*-value^*^	*P*.adj.BH^#^	*P*.adj.bon^$^	OR
***POLR3A***	4	5	0.00	0.102886	0.199012	8.72
***SUFU***	3	2	0.01	0.102886	0.231383	16.21
***LHX3***	2	1	0.02	0.218308	0.873233	21.39
***CREB3L4***	2	2	0.04	0.229436	1	10.68

*Calculation of p-value used Fisher exact test. ^#^Calculation of adjusted p-value used Benjamini and Hochberg correction. ^$^Calculation of adjusted p-value used Bonferroni correction. VUS, Variants of uncertain significance; GHD, Growth hormone deficiency; OR, odds ratio.

*POLR3A* encodes the largest subunit of RNA transcriptase III. Bi-allelic mutation in this gene is known to cause two hereditary neurological diseases, leukodystrophy, hypomyelinating 7 (4H Leukodystrophy, MIM: #607694) and Wiedemann-Rautenstrauch Syndrome (MIM: #264090). Through mutation burden analysis, four rare heterozygous and *in silico* predicted deleterious variants in *POLR3A* were significantly enriched in GHD patients without molecular diagnosis, and these variants were located in the conserved sites based on GERP++ scores (28) (**Table 4**). In addition to the peak concentration of GH in provocation test < 7ng/ml and serum IGF1 lower than 0 SD, three of the four patients showed abnormal pituitary MRIs. DISCO-S122 harbored c.200G>A(p.Arg67His) mutation with 29 CADD scores, presenting with a small pituitary and no neurohypophysis, and accompanied by small penis. Pituitary MRI found Sella turcica dysplasia accompanied by a cerebrospinal fluid hernia, small pituitary morphology and lip cleft in DISCO-S227 patient with c.1721G>T(p.Gly574Val) mutation. The CADD score for this mutation was 33. Pituitary MRI of DISCO-S596 patient with c.2672G>A(p. Arg891Gln) mutation, of which CADD score was 36, showed pituitary stalk interruption syndrome, as well as combined congenital hypothyroidism and polydipsia polyuria. The patient DISCO-S059 had a c.1676T>G(p. Phe559Cys) mutation with 24.2 CADD score, and showed a GH provocation test peak value of below 7ng/ml and decreased serum IGF1 level (**Table 4**).

**Table 4:** Information of rare VUS in genes associated with GHD and phenotype of patients.

ID	Gender	Chronological age, yrs.	Height SDS	Delayed bone age, yrs.	IGF1 SDS	Peak of GH provocation test, ng/ml	Pituitary MRI	Gene symbol	Variant type	Zygosity	Chr_position#	Ref transcript	Variant nomenclature	ExAC EAS frequency	GenomAD Exom EAS frequency	Gerp++ score	CADD score
**DISCO-S122**	M	8.83	-2.50	-2.83	-2.00	0.14	Small pituitary, no neurohypophysis	*POLR3A*	Missense	Het	Chr_10:79785498	NM_007055.3	c.200G>A (p.Arg67His)	0	0	5.18	29
**DISCO-S059**	M	4.33	-2.50	-2.33	-1.36	2.16	No obvious abnormality	*POLR3A*	Missense	Het	Chr_10:79769716	NM_007055.3	c.1676T>G (p.Phe559Cys)	0	0	5.42	24.2
**DISCO-S227**	M	9.67	-3.10	-3.67	-1.36	0.79	Sella turcica dysplasia accompanied by cerebrospinal fluid hernia, small pituitary	*POLR3A*	Missense	Het	Chr_10:79769671	NM_007055.3	c.1721G>T (p.Gly574Val)	0	0	5.72	33
**DISCO-S596**	M	11.50	-2.00	-2.50	-2.00	0.14	Pituitary stalk interruption syndrome	*POLR3A*	Missense	Het	Chr_10:79753070	NM_007055.3	c.2672G>A (p.Arg891Gln)	0	0	5.87	36
**DISCO-S150**	M	6.42	-2.31	-1.92	-1.82	0.66	No obvious abnormality	*SUFU*	Missense	Het	Chr_10:104309782	NM_016169.3	c.373A>C (p.Lys125Gln)	0	0	5.35	21.3
**DISCO-S041**	M	7.00	-3.00	-2.00	-3.30	5.02	No obvious abnormality	*SUFU*	Missense	Het	Chr_10:104357008	NM_016169.3	c.868A>G (p.Ser290Gly)	0	0	6.03	21.1
**DISCO-S085**	M	2.83	-3.00	-0.83	-0.39	5.72	No obvious abnormality	*LHX3*	Missense	Het	Chr_9:139089221	NM_014564.4	c.1159C>A (p.Pro387Thr)	0	0	4.27	25
**DISCO-S311**	M	11.33	-3.00	-4.83	-1.11	6.22	No obvious abnormality	*LHX3*	Missense	Het	Chr_9:139090764	NM_014564.4	c.611G>T (p.Arg204Leu)	0	0	3.93	28.8
**DISCO-S039**	F	9.67	-3.10	-0.67	-1.45	4.72	No obvious abnormality	*CREB3L4*	Missense	Het	Chr_1:153945476	NM_130898.3	c.665G>A (p.Arg222Lys)	0	0	5.43	36
**DISCO-S605**	F	9.41	-2.00	-0.41	-2.56	2.03	No obvious abnormality	*CREB3L4*	Frameshift	Het	Chr_1:153941539	NM_130898.3	c.312delC (p.Arg105GlyfsTer29)	0	0	–	–

^#^Chr_position was based on GRCh37. VUS, Variants of uncertain significance; F, female; M, male; Het, heterozygous; Chr, chromosome; ExAC, Exome Aggregation Consortium; CADD, Combined Annotation Dependent Depletion; gnomAD, Genome Aggregation Database.

*SUFU* encodes a negative regulatory factor in the sonic hedgehog pathway. Germline and somatic mutation of this gene can result in developmental abnormalities and tumor predisposition syndrome. In 90 GHD patients without molecular diagnosis, two patients carried rare heterozygous variants, namely, c.373A>C(p. Lys125Gln) in patient DISCO-S150 and c.868A>G(p. Ser290Gly) in patient DISCO-S041 (**Table 4**). The CADD scores for these two variants were slightly higher than 21, and both variants replaced conserved residues of the respective protein. Patient DISCO-S150 showed isolated GHD and short stature. In contrast, patient DISCO-S041 showed anemia, epiphyseal brush-like changes, reduced bone density, tooth dysplasia, skin pigmentation and thin subcutaneous fat (**Table 4**).

*LHX3* encodes a LIM domain transcription factor, which is involved in the early steps of pituitary ontogenesis. It has been reported that mutation of this gene causes combined pituitary hormone deficiency (MIM: #221750) with impaired production of GH and one or more of the other five anterior pituitary hormones through a recessive mode (29). We found two predicted deleterious *LHX3* missense variants in two patients. The c.1159C>A(p.Pro387Thr) in *LHX3* was identified in DISCOS-085, with 25 CADD score (**Table 4**). This male patient was 2 years and 10 months old, presenting with severe growth retardation. The serological examination showed a peak value of GH at 5.72 ng/ml, and IGF1 was -0.39 SDs, while imaging examination found that his bone age was delayed by 10 months and pituitary MRI had no obvious abnormality. The other patient, DISCO-S311 was an 11-year old male with short stature, and carried a heterozygous missense variants (c.611G>T[p.Arg204Leu]) in *LHX3* with high CADD score of 28.8. Serological examination showed that the peak concentration of GH were 6.22 ng/ml and 6.54 ng/ml, IGF1 was -1.11 SDs lower than normal, imaging examination showed that the bone age was delayed by 4 years and the pituitary MRI had no obvious abnormality (**Table 4**).

*CREB3L4*, located in the region of chromosome 1q21.3, codes a member of cAMP response element-binding (CREB) proteins family. The encoded protein contains 1 bZIP domain and 2 transmembrane domains. One missense variant (c.665G>A[p.Arg222Lys]) located in conserved residue with a high CADD score from DISCO-S039, and one truncating variant from DISCO-S605 (c.312delC[p.Arg105GlyfsTer29]) were found in GHD patients without molecular diagnosis (**Table 4**). Taken together, these four genes represent potential novel genes for GHD patients.

## Discussion

GHD is a rare condition characterized by the limitation of growth and development in children caused by defects in GH synthesis and secretion. However, the methods for diagnosing GHD are based mainly on clinical examinations rather than etiologically findings. In this study, we recruited 109 possible GHD patients to evaluate the consistency between clinical diagnosis and WES-based molecular testing, and how molecular testing can compensate for limitations in the clinical diagnosis of GHD. We also deciphered the molecular architecture of GHD by analyzing variants in genes biologically related with the GH axis.

Due to the wide variety of clinical symptoms of GHD and the pulsatile mode of GH secretion, there is no standard for the diagnosis of GHD (3). As a result, a certain false positive rate exists in a variety of diagnostic methods, even if combined with the measurement of serum IGF1 levels and the GH provocation test (11, 13, 30). A previous study of molecular screening of GHD in 80 Morocco patients reported that 10% patients had monogenic defect, and serologic results of 2 patients with molecular finding in this study did not meet current diagnosed criteria (31). In our cohort study, five patients with pathogenic mutations in GH-IGF1 axis-related genes did not show a phenotype that fully met the stringent clinical diagnostic criteria of GHD. This indicates that genetic testing in GHD patients is a useful tool for clinical diagnosis and genetic counseling, which can assist clinical diagnosis.

At the same time, our results indicate that results from serologic laboratory testing still have a certain possibility of false negatives. Especially patients with mutations in the same gene cannot be fully diagnosed GHD, such as *IDS* and *GHRHR*. Recent genotype-phenotype correlation study in 24 patients with *GHRHR* mutation found that the different mutation types could not fully explain the phenotypic discrepancy (32), which is consistent with our findings. In addition, the inconsistent severity of phenotypes was also observed in two *IDS* patients with the same mutation. This might be caused by variable penetrance, where the same variant in a certain gene might give rise to different manifestations and severity of the disease. The underlying mechanism of the variable penetrance might be the genetic background and modifiers, which need to be further studied using larger sample size of phenotype-genotype correlation analysis. However, these results indicates that the stringent clinical diagnostic methods have a considerable false negative rate. Therefore, we propose that precise genetic testing may be helpful to further understanding the molecular etiology and mechanism of GHD development.

Although the underlying pathogenesis of GHD developmental defects is associated with pituitary dysplasia, Sella dysplasia and GH-IGF1 axis gene mutations, most genetic causes of GHD have not been identified, which may lead a certain false-negative rate in diagnostic approach. Recently, it has been reported that some patients with Potocki-Lupski syndrome or Prader-Willi syndrome have similar phenotypes as GHD patients (33, 34), and recessive mutations in *RNPC3* could also lead to the occurrence and development of GHD (35). In our study, we found seven genes (*PTPN11*, *ROR2*, *ACAN*, *COL11A1*, *TRPS1*, *IDS*, *MMP13*) might associate with dysfunction of GH-IGF axis, indicating GHD may exist in a variety of Mendelian syndromes. Pathogenic mutations in these seven genes also might be incidental finding, which need more evidence for validation.

To further elucidate the mutational spectrum of GHD patients, we performed gene-based mutation burden analysis and identified predicted deleterious variants in 4 genes (*POLR3A*, *SUFU*, *LHX3* and *CREB3L4*) that were enriched in GHD patients, implicating that these four genes are likely novel effectors for GHD. However, the small sample size limited the power of detecting GHD-associated genes. We choose the variants in the top four genes with *p*<0.05 as the statistical trend. Recently, a multicenter retrospective study suggested that patients with 4H leukodystrophy (hypomyelination, hypodontia, and hypogonadotropic hypogonadism) may have more obvious GH secretion reduction and deficiency of various anterior pituitary hormones, such as FSH and LH (36). Our results suggest that rare heterozygous variants in *POLR3A* may lead to different degrees of recessive phenotype in patients through dose-effect models. Due to the ‘gate keeper’ role of *SUFU* in sonic hedgehog pathway, mutations in the *SUFU* gene were mostly studied in the context of susceptible tumor diseases (37). As far as we know, this is the first time that this gene is reported to be related to GHD. Previous reports found that the KO mouse model of this gene complete loss of white fat (38), which is partially consistent with the phenotype we observed in our patient. Also, a cohort study found that mutations in genes related to the hedgehog pathway were significantly enriched in patients with pituitary stalk interruption syndrome, a common pituitary change of GHD (39). Combined with our results, this suggests that the hedgehog pathway may be critical to the development of the pituitary gland. However, the specific mechanism of GHD caused by this gene requires further verification through *in vivo* and *in vitro* functional tests.

Biallelic mutations in the *LHX3* have been associated with combined pituitary hormone deficiency (MIM: #221750), characterized by impaired production of GH and other anterior pituitary hormones (29). It has been previously reported that there is variable phenotypic expressivity associated with different mutations in this gene (40, 41). Recently, Jullien et al. reported dominant pattern of *LHX3* might lead to a mild phenotype of combined pituitary hormone deficiency (41). Our findings support this new pathogenic pattern and the effect of heterozygous *LHX3* variants on the synthesis and secretion of GH. The *CREB3L4* gene is mainly expressed in prostate tissue. However, it is rarely expressed in brain tissue (42). Previous animal model studies found that male mice with *Creb3l4* knockout had spermatogenesis disorder phenotype (43). In our study, the two GHD patients with *CREB3L4* mutation were both females, and it is the first report that rare mutations of the gene are associated with human diseases. Therefore, we speculate that this gene may have sex-dependent expression characteristics, which may lead to the occurrence and development of GHD.

## Conclusion

Our study was conducted based on a relatively large cohort of GHD patients with clinical characteristics and WES data. We found the discrepancy between serologic laboratory testing and molecular diagnostic methods. In addition to known causal genes, rare variants in 4 genes related to GH synthesis and secretion were identified by mutational burden analysis. These results suggest that predicted deleterious variants are enriched in GHD patients. Therefore, we recommend providing molecular testing for all possible GHD patients.

## Data Availability Statement

The raw data supporting the conclusions of this article will be made available by the authors, without undue reservation.

## Ethics Statement

Informed consent was provided by each participant or their parents. Approval for the study was obtained from the ethics committee at the Maternal and Child Health Hospital of Guangxi Zhuang Autonomous Region (G-1-1), the Second Affiliated Hospital of Guangxi Medical University (2020-KY[0112]) and Beijing Children’s Hospital (Y-028-A-01).

## Author Contributions

All authors contributed to the article and approved the submitted version. Conceptualization: CY, SZ, CG, XF, and NW. Data curation: CY, BX, ZY, SZ, XC, XL, NW, and LL. Formal Analysis: CY, BX, ZY, SZ, YZ, LWa, and LL. Funding acquisition: SC, JZ, ZW, XF, and NW. Investigation: BC, JS, JC, CS, YS, LWe, YW, LF, BZ, and CL. Resources: CG, XF, and NW. Software: HZ and YN. Supervision: CG, XF, and NW. Visualization: HZ and CY. Writing – original draft: CY, BX, ZZ, and SZ. Writing – review and editing: CG, XF, NW, CY, SZ, and LL.

## Funding

This study received funding from the Beijing Natural Science Foundation (JQ20032 to NW and to 7191007 to ZW), National Natural Science Foundation of China (81822030 and 82072391 to NW, 81772299 and 81930068 to ZW, 81672123 and 81972037 to JZ, 82102522 to LWa), Tsinghua University-Peking Union Medical College Hospital Initiative Scientific Research Program, National Key Research and Development Program of China (2018YFC0910500 to NW and ZW), the CAMS Initiative Fund for Medical Sciences (2016-I2M-3-003 to NW, 2016-I2M-2-006 and 2017-I2M-2-001 to ZW), the Non-profit Central Research Institute Fund of Chinese Academy of Medical Sciences (No. 2019PT320025), and the Second Affiliated Hospital of Guangxi Medical University Scientific Research Program (EFYKY2020005 to XF). This study partially received funding from GeneScience Pharmaceuticals Co., Ltd (Changchun, China). The funder was not involved in the study design, collection, analysis, interpretation of data, the writing of this article or the decision to submit it for publication.

## Conflict of Interest

The authors declare that the research was conducted in the absence of any commercial or financial relationships that could be construed as a potential conflict of interest.

## Publisher’s Note

All claims expressed in this article are solely those of the authors and do not necessarily represent those of their affiliated organizations, or those of the publisher, the editors and the reviewers. Any product that may be evaluated in this article, or claim that may be made by its manufacturer, is not guaranteed or endorsed by the publisher.
