# Supplementary material for: Whole Exome Sequencing Uncovered the Genetic Architecture of Growth Hormone Deficiency Patients
Source: Front Endocrinol (Lausanne). 2021 Sep 13;12:711991. doi: 10.3389/fendo.2021.711991 (PMC8475633; doi:10.3389/fendo.2021.711991)
Supplement: Supplementary file 1 [file DataSheet_1.docx]

**Supplementary Material**

This supplemental file was intended for publication as a data supplement. The file includes three tables cited in the manuscript.

The contents include the following:

**Supplementary Methods**

**Supplementary Reference**

**Fig. S1. Disease-causing genes associated with GHD**

**Table S1. List of candidate genes associated with GH secretion and synthesis as well as related diseases**

**Table S2. Burden analysis of enriched candidate gene**

**Supplementary Methods**

**DNA extraction and exome sequencing**

For all centers, genomic DNA for 109 GHD patients (including 9 cases along with their healthy parents and 100 singletons) was extracted from peripheral blood lymphocytes. Whole-exome sequencing (WES) was performed on peripheral blood DNA for all participants. DNA samples were prepared in Illumina libraries and then underwent whole-exome capture with the SureSelect Human All Exon V5 (Agilent, USA, n= 61), SureSelect Human All Exon V6 + UTR r2 core design (Agilent, USA, n= 43), and Trueseq DNA Exome (Illumina, USA, n = 5), followed by sequencing on the Illumina HiSeq 4000 platform in 150-bp paired-end reads mode (Illumina, San Diego, CA, USA). The average sequencing depth of our cohort was 80X.

**Annotation pipeline**

The sequencing data were analyzed and annotated using an in-house developed analytical pipeline, Peking Union Medical College hospital Pipeline (PUMP) (1; 2). Paired sequences obtained from each sample were aligned to the GRCh37/hg19 human reference sequence using Burrows-Wheeler Aligner (BWA) with the MEM algorithm. BAM files were generated by Picard. Sequence reads were recalibrated by Realigner Target Creator in Genome Analysis Toolkit (GATK) and sequence variants were called by GATK Haplotype Caller. Annotation of *de novo*, compound heterozygous, and recessive inherited variants were calculated with Gemini (version 0.19.1) for *in silico* subtraction of parental variants from the proband’s variants, with accounting for read number information extracted from BAM files. Computational prediction tools (GERP++ (3), Combined Annotation Dependent Depletion (CADD)(4), SIFT(5), and Polyphen-2(6)) were used to predict the conservation and pathogenicity of candidate variants. Population frequency of each variant were obtained from publicly available databases such as the 1000 Genomes Project (<http://www.internationalgenome.org/>), the Exome variant server, NHLBI GO Exome Sequencing Project (ESP) (<http://evs.gs.washington.edu/EVS/>), the Exome Aggregation Consortium (ExAC) (<http://exac.broadinstitute.org/)>, and genome Aggregation Database (gnomAD , <http://gnomad.broadinstitute.org/>).

**Interpretation of genetic variants**

All variants were first filtered against a general population frequency of 0.01 based on 1000 Genomes, ExAC, and gnomAD databases. Intronic/UTR variants outside canonical splicing sites and synonymous variants without functional reports.

Evaluation of the pathogenicity of the variants was based on the American College of Medical Genetics and Genomics (ACMG) guidelines(7). The following categories of supportive evidence were collected for each susceptive variant:

- Very strong evidence 1 (PVS1): Protein-truncating variants (nonsense, frameshift, canonical splice sites, or start codon) in genes where loss-of-function (LoF) is a known pathogenic mechanism of the disease.
- Strong evidence 1 (PS1): Variants which result in the same amino acid changes with established pathogenic variants.
- Strong evidence 2 (PS2): Variants which arise *de novo* in a trio family where both parents are unaffected.
- Strong evidence 3 (PS3): Variants with well-designed *in vitro* or *in vivo* experiment to validate their function.
- Moderate evidence 1 (PM1): Variants located in mutational hot spot or well-validated functional domain of the proteins.
- Moderate evidence 2 (PM2): Absent or at extremely low frequency (for recessive genes) from population database (gnomAD, ExAC)
- Moderate evidence 3 (PM3): Variants with another pathogenic/likely pathogenic variant *in trans* for recessive genes.
- Moderate evidence 4 (PM4): Variants which result in changes in protein length, such as in-frame indels and stop-loss variants.
- Supporting evidence 1 (PP1): Variants co-segregated with disease in families with multiple affected members.
- Supporting evidence 2 (PP2): Missense variants in a gene in which missense variants are highly suspected to be deleterious.
- Supporting evidence 3 (PP3): Variants predicted to be deleterious by multiple *in silico* prediction tools.
- Supporting evidence 4 (PP4): Phenotypes of the patient is highly specific to the manifestation of the disease.

Then, variant pathogenicity was decided according to the existence and combination of the supportive evidence:

- Pathogenic variant: a) one very strong evidence AND one strong evidence; b) one very strong evidence AND two moderate evidence; c) one strong evidence AND three moderate evidence.
- Likely pathogenic variant: a) one very strong evidence AND one moderate evidence; b) one strong evidence AND one moderate evidence; c) three moderate evidence; d) two moderate evidence AND two supporting evidences.

After selection of pathogenic or likely pathogenic alleles, anticipated mode of inheritance associated with the identified genes was then considered. For dominant or X-linked dominant genes, a heterozygous (or hemizygous) variant is sufficient to be potentially disease-causing. For genes usually associated with an autosomal recessive disease trait inheritance, biallelic variants revealed through trio exome sequencing were required to suspect a gene to be disease-causing.

If the observed variant(s) is/are pathogenic and consistent with the expected mode of inheritance, gene-related phenotypes were compared to the patient phenotype. A positive molecular finding is defined when the phenotypic spectrum of the gene could explain the whole clinical presentation of the patient.

**Supplementary Reference**

[1] Zhao S, Zhang Y, Chen W, Li W, Wang S, Wang L, et al. Diagnostic yield and clinical impact of exome sequencing in early-onset scoliosis (EOS). *J Med Genet* (2021)58:41-47.

[2] Wang K, Zhao S, Liu B, Zhang Q, Li Y, Liu J, et al. Perturbations of BMP/TGF-beta and VEGF/VEGFR signalling pathways in non-syndromic sporadic brain arteriovenous malformations (BAVM). *J Med Genet* (2018)55:675-684.

[3] Davydov EV, Goode DL, Sirota M, Cooper GM, Sidow A, Batzoglou S. Identifying a high fraction of the human genome to be under selective constraint using GERP++. *PLoS computational biology* (2010)6:e1001025.

[4] Kircher M, Witten DM, Jain P, O'Roak BJ, Cooper GM, Shendure J. A general framework for estimating the relative pathogenicity of human genetic variants. *Nat Genet* (2014)46:310-5.

[5] Vaser R, Adusumalli S, Leng SN, Sikic M, Ng PC, SIFT missense predictions for genomes. *Nature protocols* (2016)11:1-9.

[6] Adzhubei IA, Schmidt S, Peshkin L, Ramensky VE, Gerasimova A, Bork P, et al., A method and server for predicting damaging missense mutations. *Nature methods* (2010)7:248-9.

[7] Richards S, Aziz N, Bale S, Bick D, Das S, Gastier-Foster J, et al., Standards and guidelines for the interpretation of sequence variants: a joint consensus recommendation of the American College of Medical Genetics and Genomics and the Association for Molecular Pathology. *Genet Med* (2015)17:405-24.

**Fig. S1. Disease-causing genes associated with GHD**


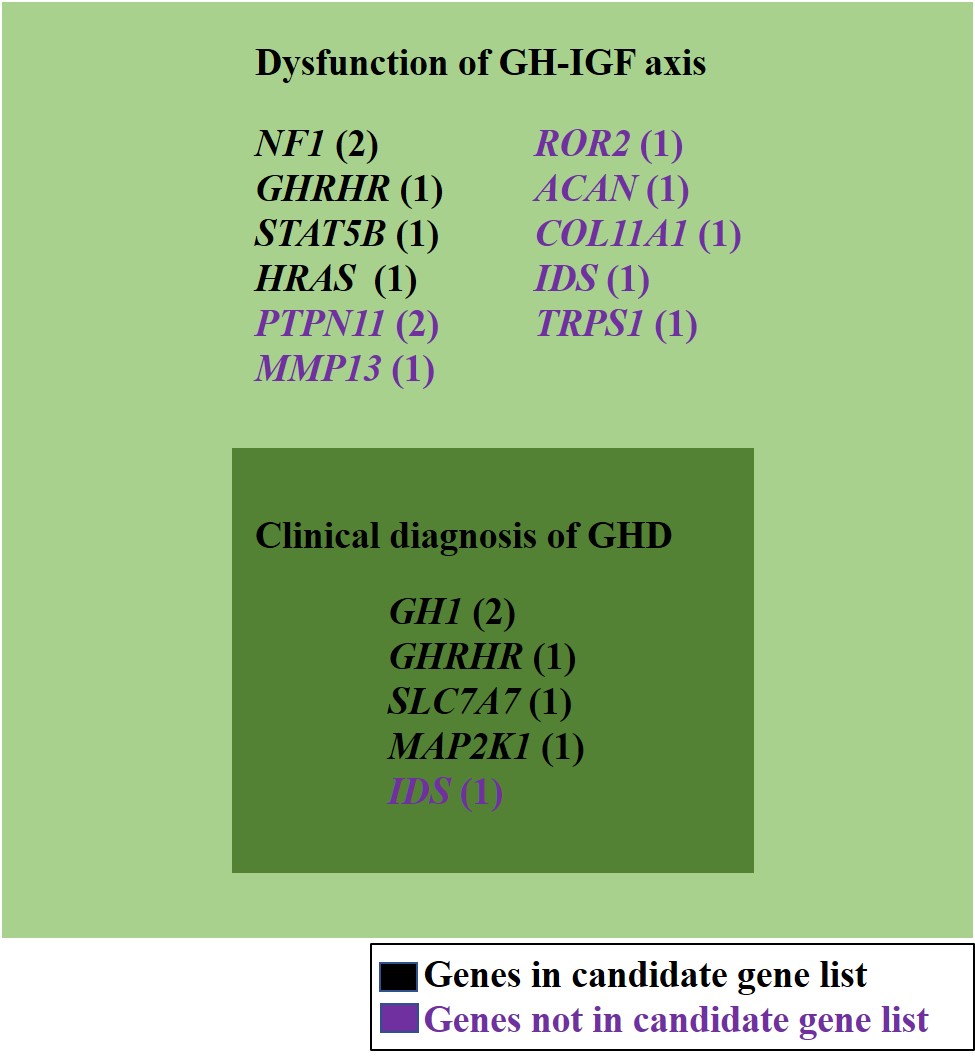


**Table S1. List of candidate genes associated with GH secretion and synthesis as well as related diseases**

| **Gene symbol** | **Evidence** |
| --- | --- |
| *LHX3* | Pituitary hormone deficiency, combined, 3, 221750 (3), Autosomal recessive |
| *FLNB* | ORPHA:56305, OMIM:108721, OMIM:272460, OMIM:150250, ORPHA:1190, ORPHA:1263, OMIM:108720, OMIM:112310, ORPHA:503 |
| *MTOR* | Mechanistic target of rapamycin kinase [KO:K07203] [EC:2.7.11.1] |
| *POLE* | ORPHA:447877, OMIM:615139, ORPHA:85173, OMIM:615083, OMIM:618336 |
| *FGF17* | ORPHA:432, ORPHA:478, OMIM:615270, OMIM:146110 |
| *FGFR1* | OMIM:101600, ORPHA:2117, OMIM:615465, ORPHA:3366, ORPHA:2645, ORPHA:3157, OMIM:147950, ORPHA:93258, OMIM:166250, OMIM:123150, ORPHA:280200, OMIM:190440, ORPHA:432, ORPHA:478, ORPHA:99798, OMIM:613001, ORPHA:2396 |
| *CTNNB1* | OMIM:114550, ORPHA:404473, ORPHA:873, OMIM:132600, OMIM:617572, ORPHA:54595, OMIM:615075, OMIM:155255, ORPHA:33402, OMIM:167000 |
| *HRAS* | HRas proto-oncogene, GTPase [KO:K02833] |
| *UCP2* | ORPHA:276556 |
| *CDON* | OMIM:614226, ORPHA:280200, ORPHA:95496 |
| *CDH23* | OMIM:601067, OMIM:601386, ORPHA:91347, ORPHA:231169, ORPHA:2965, OMIM:617540, ORPHA:96253 |
| *GLI3* | OMIM:146510, ORPHA:36, OMIM:174700, OMIM:241800, OMIM:175700, ORPHA:93322, OMIM:174200, ORPHA:380, ORPHA:672 |
| *ATF6B* | Activating transcription factor 6 beta [KO:K09049] |
| *RRM2B* | ORPHA:298, ORPHA:329336, OMIM:613077, OMIM:612075, ORPHA:480, ORPHA:254892 |
| *AKT2* | AKT serine/threonine kinase 2 [KO:K04456] [EC:2.7.11.1] |
| *SPRY4* | ORPHA:432, OMIM:615266, ORPHA:478, OMIM:146110 |
| *PIK3CB* | Phosphatidylinositol-4,5-bisphosphate 3-kinase catalytic subunit beta [KO:K00922] [EC:2.7.1.153] |
| *VPS13B* | OMIM:216550, ORPHA:193 |
| *PITX2* | OMIM:137600, ORPHA:782, ORPHA:708, OMIM:180500, OMIM:180550 |
| *PLCB3* | Phospholipase C beta 3 [KO:K05858] [EC:3.1.4.11] |
| *CREB3L4* | cAMP responsive element binding protein 3 like 4 [KO:K09048] |
| *SUFU* | ORPHA:36, OMIM:607174, OMIM:617757, OMIM:155255, ORPHA:280200, ORPHA:377, OMIM:109400, ORPHA:2495 |
| *SSTR3* | Somatostatin receptor 3 [KO:K04219] |
| *SLC7A7* | ORPHA:470, OMIM:222700 |
| *PIK3R3* | Phosphoinositide-3-kinase regulatory subunit 3 [KO:K02649] |
| *POU1F1* | Pituitary hormone deficiency, combined, 1, 613038 (3), Autosomal recessive, Autosomal dominant |
| *KMT2A* | ORPHA:199, OMIM:605130, ORPHA:319182 |
| *SHC1* | SHC adaptor protein 1 [KO:K06279] |
| *DMPK* | ORPHA:273, OMIM:160900 |
| *ABCC8* | ORPHA:99885, ORPHA:276575, OMIM:256450, OMIM:240800, OMIM:610374, ORPHA:79134, ORPHA:99886, OMIM:125853, OMIM:606176, ORPHA:552 |
| *FANCA* | Fanconi anemia, complementation group A, 227650 |
| *SOS2* | SOS Ras/Rho guanine nucleotide exchange factor 2 [KO:K03099] |
| *NSMF* | ORPHA:432, ORPHA:478, OMIM:614838 |
| *ADCY6* | Adenylate cyclase 6 [KO:K08046] [EC:4.6.1.1] |
| *UBE3B* | Kaufman oculocerebrofacial syndrome, 244450 |
| *ADCY4* | Adenylate cyclase 4 [KO:K08044] [EC:4.6.1.1] |
| *ADCY9* | Adenylate cyclase 9 [KO:K08049] [EC:4.6.1.1] |
| *CHD7* | ORPHA:432, OMIM:612370, ORPHA:138, ORPHA:478, OMIM:214800, ORPHA:39041 |
| *TBCK* | OMIM:616900, ORPHA:488632 |
| *GLI2* | OMIM:610829, ORPHA:280200, ORPHA:95494, OMIM:615849 |
| *PLCG2* | Phospholipase C gamma 2 [KO:K05859] [EC:3.1.4.11] |
| *TMEM67* | OMIM:216360, OMIM:613550, OMIM:602152, OMIM:610688, OMIM:615991, ORPHA:475, OMIM:607361, ORPHA:564, ORPHA:1454 |
| *MAGEL2* | OMIM:176270, OMIM:615547 |
| *HERC2* | OMIM:176270, OMIM:615516 |
| *NODAL* | OMIM:270100, ORPHA:280200 |
| *SHH* | ORPHA:93321, OMIM:611638, ORPHA:3332, OMIM:269160, ORPHA:93405, ORPHA:280200, OMIM:147250, OMIM:142945 |
| *SHOC2* | Noonan syndrome-like with loose anagen hair, 607721 |
| *HS6ST1* | ORPHA:432, ORPHA:478, OMIM:614880 |
| *LEPR* | ORPHA:179494,OMIM:614963 |
| *MAPK9* | Mitogen-activated protein kinase 9 [KO:K04440] [EC:2.7.11.24] |
| *ATF4* | Activating transcription factor 4 [KO:K04374] |
| *BRAF* | OMIM:211980, ORPHA:54595, ORPHA:1340, OMIM:613706, ORPHA:500, OMIM:613707, OMIM:115150, OMIM:163950 |
| *TBX2* | OMIM:618223 |
| *IGFALS* | Insulin like growth factor binding protein acid labile subunit [KO:K17256] |
| *PPARG* | ORPHA:79083, ORPHA:528, OMIM:125853, OMIM:604367, OMIM:601665 |
| *PLCB1* | Phospholipase C beta 1 [KO:K05858] [EC:3.1.4.11] |
| *ADCY2* | Adenylate cyclase 2 [KO:K08042] [EC:4.6.1.1] |
| *BCAR1* | BCAR1 scaffold protein, Cas family member [KO:K05726] |
| *GUCY2D* | PubMed: 9268092; OMIM:600179 |
| *PIK3R2* | Phosphoinositide-3-kinase regulatory subunit 2 [KO:K02649] |
| *TRAPPC11* | ORPHA:869, ORPHA:369840, OMIM:615356, ORPHA:369847 |
| *MAP3K1* | Mitogen-activated protein kinase kinase kinase 1 [KO:K04416] [EC:2.7.11.25] |
| *CACNA1D* | Calcium voltage-gated channel subunit alpha1 D [KO:K04851] |
| *ALMS1* | ORPHA:64, OMIM:203800 |
| *PLCB2* | Phospholipase C beta 2 [KO:K05858] [EC:3.1.4.11] |
| *CACNA1S* | Calcium voltage-gated channel subunit alpha1 S [KO:K04857] |
| *ITPR3* | Inositol 1,4,5-trisphosphate receptor type 3 [KO:K04960] |
| *NDN* | OMIM:176270 |
| *JAK2* | Janus kinase 2 [KO:K04447] [EC:2.7.10.2] |
| *EDA* | OMIM:305100, ORPHA:181, ORPHA:99798, OMIM:313500 |
| *GH1* | Growth hormone deficiency, isolated, type IA, 262400 (3), Autosomal recessive; Growth hormone deficiency, isolated, type IB, 612781 (3); Growth hormone deficiency, isolated, type II, 173100 (3), Autosomal dominant; Kowarski syndrome, 262650 (3), Autosomal recessive |
| *FANCI* | OMIM:609053, ORPHA:84 |
| *IGF1* | Insulin like growth factor 1 [KO:K05459] |
| *KCNJ11* | ORPHA:99885, ORPHA:276580, ORPHA:79134, ORPHA:99886, OMIM:125853, OMIM:606176, OMIM:616329, OMIM:610582, OMIM:601820, ORPHA:552 |
| *SLC29A3* | ORPHA:1782, OMIM:602782, ORPHA:168569 |
| *SSTR2* | Somatostatin receptor 2 [KO:K04218] |
| *CACNA1F* | Calcium voltage-gated channel subunit alpha1 F [KO:K04853] |
| *SSTR5* | Somatostatin receptor 5 [KO:K04221] |
| *CCDC141* | ORPHA:478, OMIM:146110 |
| *ADNP* | OMIM:615873, ORPHA:404448 |
| *GHRHR* | Growth hormone deficiency, isolated, type IV, 618157 (3) |
| *EP300* | E1A binding protein p300 [KO:K04498] [EC:2.3.1.48] |
| *GHR* | Growth hormone insensitivity, partial, 604271 (3), Autosomal dominant; Hypercholesterolemia, familial, modifier of, 143890 (3), Autosomal dominant; Increased responsiveness to growth hormone, 604271 (3), Autosomal dominant; Laron dwarfism, 262500 (3), Autosomal recessive |
| *RBM28* | OMIM:612079, ORPHA:157954 |
| *IGSF1* | Hypothyroidism, central, and testicular enlargement, 300888, XLR |
| *IRS2* | Insulin receptor substrate 2 [KO:K07187] |
| *DCC* | OMIM:617542, OMIM:133239, ORPHA:238722, ORPHA:478, OMIM:157600, ORPHA:2744 |
| *WDR11* | ORPHA:432, OMIM:614858, ORPHA:478, ORPHA:95496, OMIM:146110 |
| *ITPR2* | Inositol 1,4,5-trisphosphate receptor type 2 [KO:K04959] |
| *PNPLA6* | OMIM:612020, OMIM:245800, OMIM:215470, ORPHA:139480, ORPHA:2377, OMIM:275400, ORPHA:1173, ORPHA:1180 |
| *PTCH1* | ORPHA:77301, OMIM:605462, ORPHA:280200, ORPHA:377, OMIM:610828, OMIM:109400 |
| *CREBBP* | CREB binding protein [KO:K04498] [EC:2.3.1.48] |
| *GNAS* | GNAS complex locus [KO:K04632] |
| *AAAS* | ORPHA:869, OMIM:231550 |
| *ADAT3* | ORPHA:363528, OMIM:615286 |
| *ADCY1* | Adenylate cyclase 1 [KO:K08041] [EC:4.6.1.1] |
| *ADCY10* | Adenylate cyclase 10 [KO:K11265] [EC:4.6.1.1] |
| *ADCY3* | Adenylate cyclase 3 [KO:K08043] [EC:4.6.1.1] |
| *ADCY5* | Adenylate cyclase 5 [KO:K08045] [EC:4.6.1.1] |
| *ADCY7* | Adenylate cyclase 7 [KO:K08047] [EC:4.6.1.1] |
| *ADCY8* | Adenylate cyclase 8 [KO:K08048] [EC:4.6.1.1] |
| *AFF4* | OMIM:616368, ORPHA:444077 |
| *AGPAT2* | ORPHA:528, OMIM:608594 |
| *AIP* | ORPHA:99725, ORPHA:963, ORPHA:2965, OMIM:102200, OMIM:219090 |
| *AKT1* | AKT serine/threonine kinase 1 [KO:K04456] [EC:2.7.11.1] |
| *AKT3* | AKT serine/threonine kinase 3 [KO:K04456] [EC:2.7.11.1] |
| *ANOS1* | OMIM:308700, ORPHA:432, ORPHA:478 |
| *ARNT2* | ORPHA:3157, OMIM:615926 |
| *ATF2* | Activating transcription factor 2 [KO:K04450] |
| *B3GLCT* | ORPHA:709, OMIM:261540 |
| *BMP4* | ORPHA:199306, OMIM:600625, ORPHA:139471, OMIM:607932 |
| *BSCL2* | ORPHA:363400, ORPHA:100998, ORPHA:139536, OMIM:269700, ORPHA:528, OMIM:270685, OMIM:600794, OMIM:615924 |
| *BTK* | Agammaglobulinemia, X-linked 1, 300755 (3), X-linked recessive; Isolated growth hormone deficiency, type III, with agammaglobulinemia, 307200 (3), X-linked recessive |
| *CACNA1C* | Calcium voltage-gated channel subunit alpha1 C [KO:K04850] |
| *CAV1* | OMIM:612526, ORPHA:220402, ORPHA:528, OMIM:615343, OMIM:606721, ORPHA:220393 |
| *CAVIN1* | OMIM:613327, ORPHA:528 |
| *CDKN1C* | ORPHA:436144, OMIM:130650, ORPHA:85173, OMIM:614732 |
| *CEP57* | OMIM:614114, ORPHA:1052 |
| *CREB1* | cAMP responsive element binding protein 1 [KO:K05870] |
| *CREB3* | cAMP responsive element binding protein 3 [KO:K09048] |
| *CREB3L1* | cAMP responsive element binding protein 3 like 1 [KO:K09048] |
| *CREB3L2* | cAMP responsive element binding protein 3 like 2 [KO:K09048] |
| *CREB3L3* | cAMP responsive element binding protein 3 like 3 [KO:K09048] |
| *CREB5* | cAMP responsive element binding protein 5 [KO:K09047] |
| *CRK* | CRK proto-oncogene, adaptor protein [KO:K04438] |
| *CRKL* | CRK like proto-oncogene, adaptor protein [KO:K04438] |
| *CSH1* | Chorionic somatomammotropin hormone 1 [KO:K05438] |
| *CSH2* | Chorionic somatomammotropin hormone 2 [KO:K05438] |
| *DCAF17* | OMIM:241080, ORPHA:3464 |
| *DLL1* | ORPHA:280200 |
| *DUSP6* | ORPHA:432, OMIM:615269, ORPHA:478, OMIM:146110 |
| *EIF2S3* | ORPHA:85282, OMIM:300148 |
| *FEZF1* | ORPHA:478, OMIM:616030, OMIM:146110 |
| *FGF8* | ORPHA:432, ORPHA:478, OMIM:612702, ORPHA:280200 |
| *FLRT3* | ORPHA:478, OMIM:615271, OMIM:146110 |
| *FOS* | Fos proto-oncogene, AP-1 transcription factor subunit [KO:K04379] |
| *FSHB* | ORPHA:52901, OMIM:229070 |
| *GH2* | Growth hormone 2 [KO:K05438] |
| *GHRH* | Gigantism due to GHRF hypersecretion (1); ?Isolated growth hormone deficiency due to defect in GHRF (1) |
| *GHRL* | Ghrelin and obestatin prepropeptide [KO:K05254] |
| *GHSR* | Growth hormone deficiency, isolated partial, 615925 (3), Autosomal recessive, Autosomal dominant |
| *GMNN* | OMIM:616835, ORPHA:2554 |
| *GMPPA* | ORPHA:869, OMIM:615510 |
| *GNA11* | G protein subunit alpha 11 [KO:K04635] |
| *GNAI1* | G protein subunit alpha i1 [KO:K04630] |
| *GNAI2* | G protein subunit alpha i2 [KO:K04630] |
| *GNAI3* | G protein subunit alpha i3 [KO:K04630] |
| *GNAQ* | G protein subunit alpha q [KO:K04634] |
| *GPR101* | OMIM:300942, OMIM:300943, ORPHA:963 |
| *GRB2* | Growth factor receptor bound protein 2 [KO:K04364] |
| *GSK3B* | Glycogen synthase kinase 3 beta [KO:K03083] [EC:2.7.11.26] |
| *H19* | OMIM:180860, ORPHA:654, OMIM:130650, ORPHA:2128, OMIM:194071, OMIM:194070 |
| *HESX1* | Growth hormone deficiency with pituitary anomalies, 182230 (3), Autosomal recessive, Autosomal dominant; Pituitary hormone deficiency, combined, 5, 182230 (3), Autosomal recessive, Autosomal dominant; Septooptic dysplasia, 182230 (3), Autosomal recessive, Autosomal dominant |
| *IARS2* | ?Cataracts, growth hormone deficiency, sensory neuropathy, sensorineural hearing loss, and skeletal dysplasia, 616007 (3), Autosomal recessive |
| *IFT57* | ?Orofaciodigital syndrome XVIII, 617927 |
| *IGF2* | OMIM:180860, OMIM:130650, ORPHA:2128, OMIM:194070, OMIM:616489 |
| *IGFBP3* | Insulin like growth factor binding protein 3 [KO:K10138] |
| *IL17RD* | ORPHA:478, OMIM:615267, OMIM:146110 |
| *IPW* | OMIM:176270 |
| *IRS1* | Insulin receptor substrate 1 [KO:K16172] |
| *IRS4* | Insulin receptor substrate 4 [KO:K17446] |
| *ITPR1* | Inositol 1,4,5-trisphosphate receptor type 1 [KO:K04958] |
| *JUNB* | JunB proto-oncogene, AP-1 transcription factor subunit [KO:K09028] |
| *KATNIP* | OMIM:616784, ORPHA:475 |
| *KISS1R* | OMIM:176400, ORPHA:432, ORPHA:478, OMIM:614837 |
| *KRAS* | KRAS proto-oncogene, GTPase [KO:K07827] |
| *LEP* | OMIM:614962, ORPHA:66628 |
| *LHX4* | Pituitary hormone deficiency, combined, 4, 262700 (3), Autosomal dominant |
| *MAP2K1* | Mitogen-activated protein kinase kinase 1 [KO:K04368] [EC:2.7.12.2] |
| *MAP2K2* | Mitogen-activated protein kinase kinase 2 [KO:K04369] [EC:2.7.12.2] |
| *MAP2K3* | Mitogen-activated protein kinase kinase 3 [KO:K04432] [EC:2.7.12.2] |
| *MAP2K4* | Mitogen-activated protein kinase kinase 4 [KO:K04430] [EC:2.7.12.2] |
| *MAP2K6* | Mitogen-activated protein kinase kinase 6 [KO:K04433] [EC:2.7.12.2] |
| *MAPK1* | Mitogen-activated protein kinase 1 [KO:K04371] [EC:2.7.11.24] |
| *MAPK10* | Mitogen-activated protein kinase 10 [KO:K04440] [EC:2.7.11.24] |
| *MAPK11* | Mitogen-activated protein kinase 11 [KO:K04441] [EC:2.7.11.24] |
| *MAPK12* | Mitogen-activated protein kinase 12 [KO:K04441] [EC:2.7.11.24] |
| *MAPK13* | Mitogen-activated protein kinase 13 [KO:K04441] [EC:2.7.11.24] |
| *MAPK14* | Mitogen-activated protein kinase 14 [KO:K04441] [EC:2.7.11.24] |
| *MAPK3* | Mitogen-activated protein kinase 3 [KO:K04371] [EC:2.7.11.24] |
| *MAPK8* | Mitogen-activated protein kinase 8 [KO:K04440] [EC:2.7.11.24] |
| *MARS2* | ORPHA:314603, OMIM:611390, OMIM:616430 |
| *MCM4* | PubMed: 22499342 |
| *MEN1* | ORPHA:99879, ORPHA:99725, OMIM:131100, ORPHA:97279, ORPHA:2965, ORPHA:652 |
| *MKRN3* | OMIM:176270, OMIM:615346 |
| *MKRN3-AS1* | OMIM:176270 |
| *NF2* | ORPHA:637, OMIM:101000, OMIM:607174, ORPHA:2495, OMIM:162091 |
| *NFKB2* | ORPHA:1572, ORPHA:293978, OMIM:615577 |
| *NPAP1* | OMIM:176270 |
| *NRAS* | NRAS proto-oncogene, GTPase [KO:K07828] |
| *OTX2* | Microphthalmia, syndromic 5, 610125 (3), Autosomal dominant; Pituitary hormone deficiency, combined, 6, 613986 (3), Autosomal dominant; Retinal dystrophy, early-onset, with or without pituitary dysfunction, 610125 (3), Autosomal dominant |
| *PCSK1* | OMIM:600955, ORPHA:71528 |
| *PDE4D* | ORPHA:439822, OMIM:614613, ORPHA:280651, ORPHA:950 |
| *PDGFB* | OMIM:213600, OMIM:607174, OMIM:615483, ORPHA:1980, ORPHA:31112, ORPHA:2495 |
| *PIK3CA* | Phosphatidylinositol-4,5-bisphosphate 3-kinase catalytic subunit alpha [KO:K00922] [EC:2.7.1.153] |
| *PIK3CD* | Phosphatidylinositol-4,5-bisphosphate 3-kinase catalytic subunit delta [KO:K00922] [EC:2.7.1.153] |
| *PIK3R1* | Phosphoinositide-3-kinase regulatory subunit 1 [KO:K02649] |
| *PLCB4* | Phospholipase C beta 4 [KO:K05858] [EC:3.1.4.11] |
| *PLCG1* | Phospholipase C gamma 1 [KO:K01116] [EC:3.1.4.11] |
| *POLR3A* | OMIM:607694, ORPHA:3455, ORPHA:88637, OMIM:264090, ORPHA:447896 |
| *POMC* | ORPHA:71526, OMIM:609734, OMIM:601665 |
| *POU3F4* | ORPHA:1435, OMIM:304400 |
| *PREPL* | ORPHA:163690, ORPHA:163693, OMIM:616224 |
| *PRKACA* | Protein kinase cAMP-activated catalytic subunit alpha [KO:K04345] [EC:2.7.11.11] |
| *PRKACB* | Protein kinase cAMP-activated catalytic subunit beta [KO:K04345] [EC:2.7.11.11] |
| *PRKACG* | Protein kinase cAMP-activated catalytic subunit gamma [KO:K04345] [EC:2.7.11.11] |
| *PRKAR1A* | OMIM:160980, ORPHA:615, ORPHA:280651, OMIM:255960, ORPHA:189439, ORPHA:950, OMIM:610489, ORPHA:1359, ORPHA:520, OMIM:101800 |
| *PRKCA* | Protein kinase C alpha [KO:K02677] [EC:2.7.11.13] |
| *PRKCB* | Protein kinase C beta [KO:K19662] [EC:2.7.11.13] |
| *PRKCG* | Protein kinase C gamma [KO:K19663] [EC:2.7.11.13] |
| *PROK2* | ORPHA:432, ORPHA:478, OMIM:610628 |
| *PROKR2* | ORPHA:432, ORPHA:3157, OMIM:244200, ORPHA:478, ORPHA:95496 |
| *PROP1* | Pituitary hormone deficiency, combined, 2, 262600 (3), Autosomal recessive |
| *PTK2* | Protein tyrosine kinase 2 [KO:K05725] [EC:2.7.10.2] |
| *PWAR1* | OMIM:176270 |
| *PWRN1* | OMIM:176270 |
| *RAF1* | Raf-1 proto-oncogene, serine/threonine kinase [KO:K04366] [EC:2.7.11.1] |
| *RFWD3* | OMIM:617784, ORPHA:84 |
| *RNPC3* | ?Growth hormone deficiency, isolated, type V, 618160 (3), Autosomal recessive |
| *SEMA3A* | ORPHA:478, OMIM:614897 |
| *SEMA3E* | ORPHA:138, OMIM:214800, OMIM:146110 |
| *SHC2* | SHC adaptor protein 2 [KO:K17447] |
| *SHC3* | SHC adaptor protein 3 [KO:K17448] |
| *SHC4* | SHC adaptor protein 4 [KO:K17449] |
| *SIN3A* | ORPHA:94065, OMIM:613406 |
| *SIX3* | OMIM:157170, OMIM:269160, ORPHA:280200 |
| *SMARCB1* | OMIM:609322, OMIM:614608, ORPHA:1465, ORPHA:99966, ORPHA:2495, OMIM:162091 |
| *SMC1A* | ORPHA:199, ORPHA:319182, OMIM:300590 |
| *SNORD115-1* | OMIM:176270 |
| *SNORD116-1* | OMIM:176270 |
| *SNRPN* | OMIM:176270, OMIM:209850 |
| *SOCS1* | Suppressor of cytokine signaling 1 [KO:K04694] |
| *SOCS2* | Suppressor of cytokine signaling 2 [KO:K04695] |
| *SOCS3* | Suppressor of cytokine signaling 3 [KO:K04696] |
| *SOS1* | SOS Ras/Rac guanine nucleotide exchange factor 1 [KO:K03099] |
| *SOX10* | OMIM:613266, ORPHA:897, ORPHA:478, OMIM:611584, OMIM:609136, ORPHA:895, ORPHA:163746 |
| *SOX3* | Mental retardation, X-linked, with isolated growth hormone deficiency, 300123 (3); Panhypopituitarism, X-linked, 312000 (3), X-linked |
| *SRD5A3* | ORPHA:324737, OMIM:612379, OMIM:612713 |
| *SST* | Somatostatin [KO:K05237] |
| *SSTR1* | Somatostatin receptor 1 [KO:K04217] |
| *STAT1* | Signal transducer and activator of transcription 1 [KO:K11220] |
| *STAT3* | Signal transducer and activator of transcription 3 [KO:K04692] |
| *STAT5A* | Signal transducer and activator of transcription 5A [KO:K11223] |
| *STAT5B* | Growth hormone insensitivity with immunodeficiency, 245590 (3); Leukemia, acute promyelocytic, somatic, 102578 (3) |
| *STX16* | ORPHA:94089, OMIM:603233 |
| *TACR3* | ORPHA:432, ORPHA:478, OMIM:614840 |
| *TBCE* | OMIM:241410, OMIM:244460, ORPHA:2323, OMIM:617207, ORPHA:93324, ORPHA:496756 |
| *TBX19* | ORPHA:199296, OMIM:201400 |
| *TGIF1* | OMIM:142946, ORPHA:280200 |
| *THOC2* | ORPHA:457240, OMIM:300957 |
| *TMCO1* | OMIM:213980, ORPHA:1394 |
| *TP53* | OMIM:151623, OMIM:260500, OMIM:114480, ORPHA:3318, ORPHA:524, OMIM:137800, OMIM:114550, OMIM:618165, ORPHA:1333, OMIM:607107, OMIM:260350, ORPHA:2807, OMIM:202300, ORPHA:145, ORPHA:1501, OMIM:259500, ORPHA:668 |
| *TP63* | ORPHA:199306, OMIM:603543, OMIM:129400, ORPHA:978, OMIM:103285, OMIM:106260, ORPHA:1896, ORPHA:93930, ORPHA:199302, ORPHA:1071, ORPHA:2440, ORPHA:69085, OMIM:605289, OMIM:604292, ORPHA:141291 |
| *TRNL1* | OMIM:540000, ORPHA:255210, ORPHA:551, OMIM:545000, ORPHA:225, ORPHA:663, ORPHA:550, ORPHA:480 |
| *WASHC5* | ORPHA:7, OMIM:603563, OMIM:220210, ORPHA:100989 |
| *WDR4* | OMIM:618347, ORPHA:2065, OMIM:618346 |
| *YY1* | ORPHA:506358, OMIM:617557 |
| *ZIC2* | ORPHA:280200, OMIM:609637 |
| *ZNF148* | OMIM:617260 |

Abbreviation: OMIM, Online Mendelian Inheritance in Man; KO, KEGG Orthology; ORPHA, Orphanet database.

**Table S2. Burden analysis of enriched candidate gene**

| Gene | Case (n=90) | Control (n=942) | *P* value ^*^ | *P*.adj.BH ^#^ | *P*.adj.bon ^$^ |
| --- | --- | --- | --- | --- | --- |
| *POLR3A* | 4 | 5 | 0.004854 | 0.102886 | 0.199012 |
| *SUFU* | 3 | 2 | 0.005643 | 0.102886 | 0.231383 |
| *LHX3* | 2 | 1 | 0.021298 | 0.218308 | 0.873233 |
| *CREB3L4* | 2 | 2 | 0.040187 | 0.229436 | 1 |
| *FANCA* | 2 | 3 | 0.063216 | 0.229436 | 1 |
| *HS6ST1* | 1 | 0 | 0.087209 | 0.229436 | 1 |
| *CEP57* | 1 | 0 | 0.087209 | 0.229436 | 1 |
| *STAT3* | 1 | 0 | 0.087209 | 0.229436 | 1 |
| *FGF17* | 1 | 0 | 0.087209 | 0.229436 | 1 |
| *CTNNB1* | 1 | 0 | 0.087209 | 0.229436 | 1 |
| *SSTR5* | 1 | 0 | 0.087209 | 0.229436 | 1 |
| *GMNN* | 1 | 0 | 0.087209 | 0.229436 | 1 |
| *MARS2* | 1 | 0 | 0.087209 | 0.229436 | 1 |
| *RFWD3* | 1 | 0 | 0.087209 | 0.229436 | 1 |
| *ABCC8* | 2 | 4 | 0.089536 | 0.229436 | 1 |
| *POLE* | 3 | 12 | 0.135753 | 0.327404 | 1 |
| *RRM2B* | 1 | 1 | 0.16689 | 0.360132 | 1 |
| *PRKAR1A* | 1 | 1 | 0.16689 | 0.360132 | 1 |
| *CDH23* | 5 | 27 | 0.19032 | 0.390157 | 1 |
| *RBM28* | 1 | 2 | 0.239686 | 0.427267 | 1 |
| *SLC7A7* | 1 | 2 | 0.239686 | 0.427267 | 1 |
| *MAGEL2* | 1 | 2 | 0.239686 | 0.427267 | 1 |
| *KMT2A* | 1 | 3 | 0.306186 | 0.502145 | 1 |
| *SHC1* | 1 | 3 | 0.306186 | 0.502145 | 1 |
| *NSMF* | 1 | 4 | 0.366929 | 0.557188 | 1 |
| *DMPK* | 1 | 3 | 0.366929 | 0.557188 | 1 |
| *MTOR* | 1 | 5 | 0.422407 | 0.558667 | 1 |
| *ADCY6* | 1 | 5 | 0.422407 | 0.558667 | 1 |
| *TRAPPC11* | 1 | 5 | 0.422407 | 0.558667 | 1 |
| *CACNA1C* | 1 | 5 | 0.422407 | 0.558667 | 1 |
| *PLCG1* | 1 | 6 | 0.473073 | 0.606125 | 1 |
| *ALMS1* | 1 | 8 | 0.561585 | 0.657857 | 1 |
| *CHD7* | 1 | 8 | 0.561585 | 0.657857 | 1 |
| *TBCK* | 1 | 8 | 0.561585 | 0.657857 | 1 |
| *FLNB* | 1 | 21 | 0.713585 | 0.812694 | 1 |
| *PLCB2* | 1 | 12 | 1 | 1 | 1 |
| *ITPR3* | 1 | 12 | 1 | 1 | 1 |
| *VPS13B* | 2 | 22 | 1 | 1 | 1 |
| *UBE3B* | 1 | 15 | 1 | 1 | 1 |
| *CACNA1D* | 1 | 10 | 1 | 1 | 1 |

* Calculation of *p*-value used Fisher exact test. # Calculation of adjusted *p*-value used Benjamini and Hochberg correction. $ Calculation of adjusted *p*-value used Bonferroni correction.
